# Supplementary material for: Nanoscale optical nonreciprocity with nonlinear metasurfaces
Source: Nat Commun. 2024 Jun 13;15:5077. doi: 10.1038/s41467-024-49436-1 (PMC11176174; doi:10.1038/s41467-024-49436-1)
Supplement: Supplementary file 1 — Supplementary Information [file 41467_2024_49436_MOESM1_ESM.pdf]

Supplementary Information

Nanoscale optical nonreciprocity with nonlinear metasurfaces

Aditya Tripathi[1], Chibuzor Fabian Ugwu[2], Viktar S. Asadchy[3,4], Ivan Kravchenko[5], Shanhui Fan[3], Yuri Kivshar[1], Jason Valentine[2], and Sergey S. Kruk[1,6,*]

[1]*Nonlinear Physics Centre, Research School of Physics, Australian National University, Canberra ACT 2601, Australia*
[2]*Department of Mechanical Engineering, Vanderbilt University, Nashville, Tennessee 37212, United States*
[3]*Ginzton Laboratory, Department of Electrical Engineering, Stanford University, Stanford, 94305, CA, United States*
[4]*Department of Electronics and Nanoengineering, Aalto University, Espoo 02150, Finland*
[5]*Center for Nanophase Materials Sciences, Oak Ridge National Laboratory, Oak Ridge, Tennessee 37831, USA*
[6]*Department of Physics, Paderborn University, Paderborn 33098, Germany*

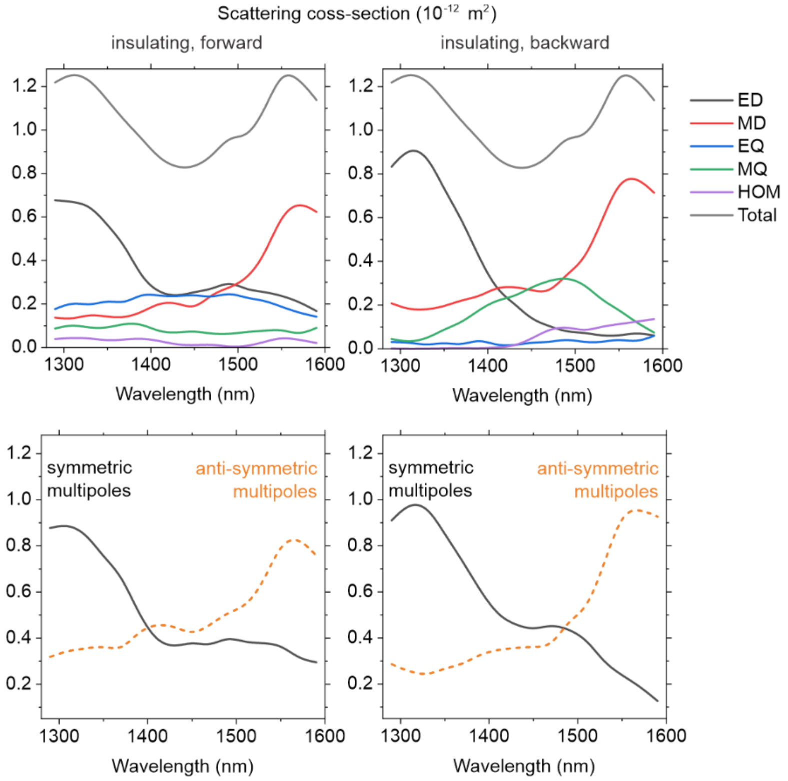

Figure S1. Top :theoretically calculated multipolar composition of scattering of the Si-VO_2 unit cell for the insulation VO2 phase in forward direction (top, left) and in backward direction (top, right). Bottom: sum of symmetric (ED, MQ) vs anti-symmetric multipoles (MD, EQ) in forward (bottom, left) and backward (bottom, right) directions.

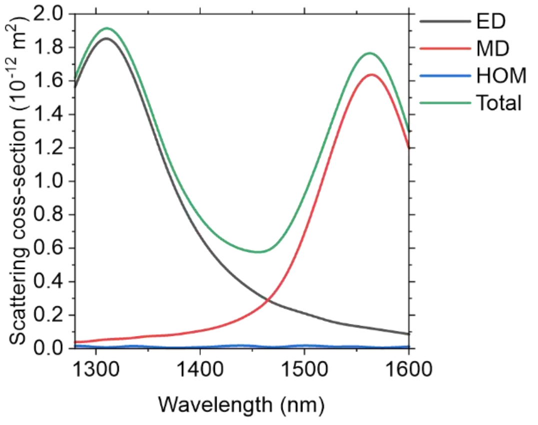

Figure S2. Theoretically calculated multipolar composition of Si unit cell in the absence of the VO2 film. Fully symmetric design results in identical forward and backward multipolar composition. In the absence of VO2 EQ and MQ contributions are negligibly small.

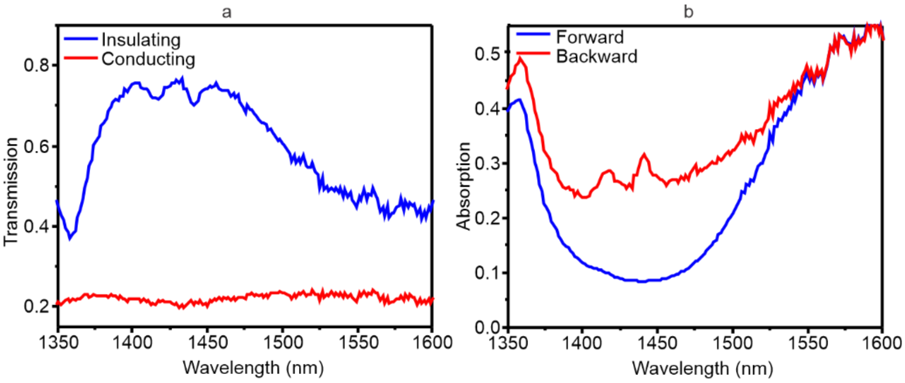

Figure S3. Experimental measurement of (a) transmission for insulating (blue) and conducting (green) metasurface and (b) absorption for forward (blue) and backward (red) illuminations.

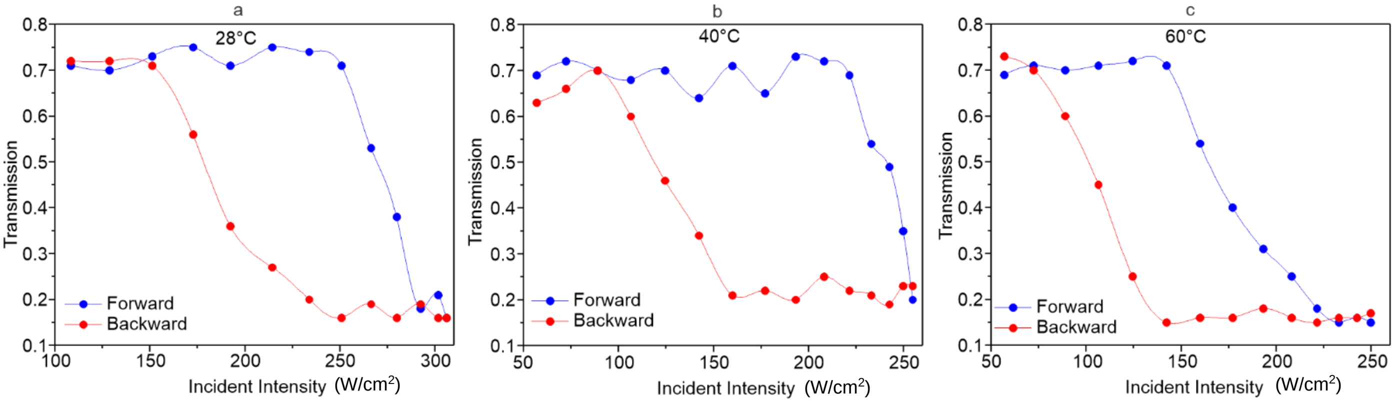

Figure S4. Experimental transmission for two opposite directions of illumination at 1470 nm wavelength as a function of an increasing intensity of light at a bias temperature of (a) 28ºC, (b) 40ºC and (c) 60ºC.

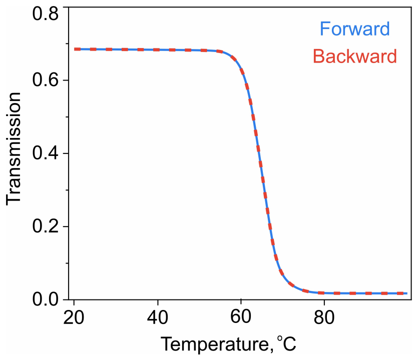

Figure S5. Theoretical calculation of transmittance as a function of temperature (heating) for low incident power.

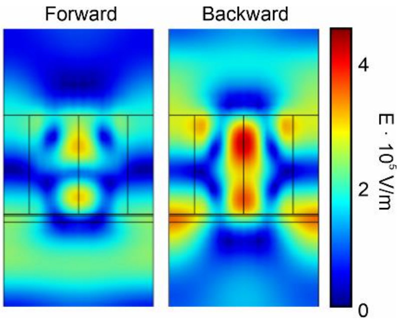

Figure S6. Theoretical calculation of electric field distributions inside the metasurface for forward and backward illumination at 1450 nm wavelength.

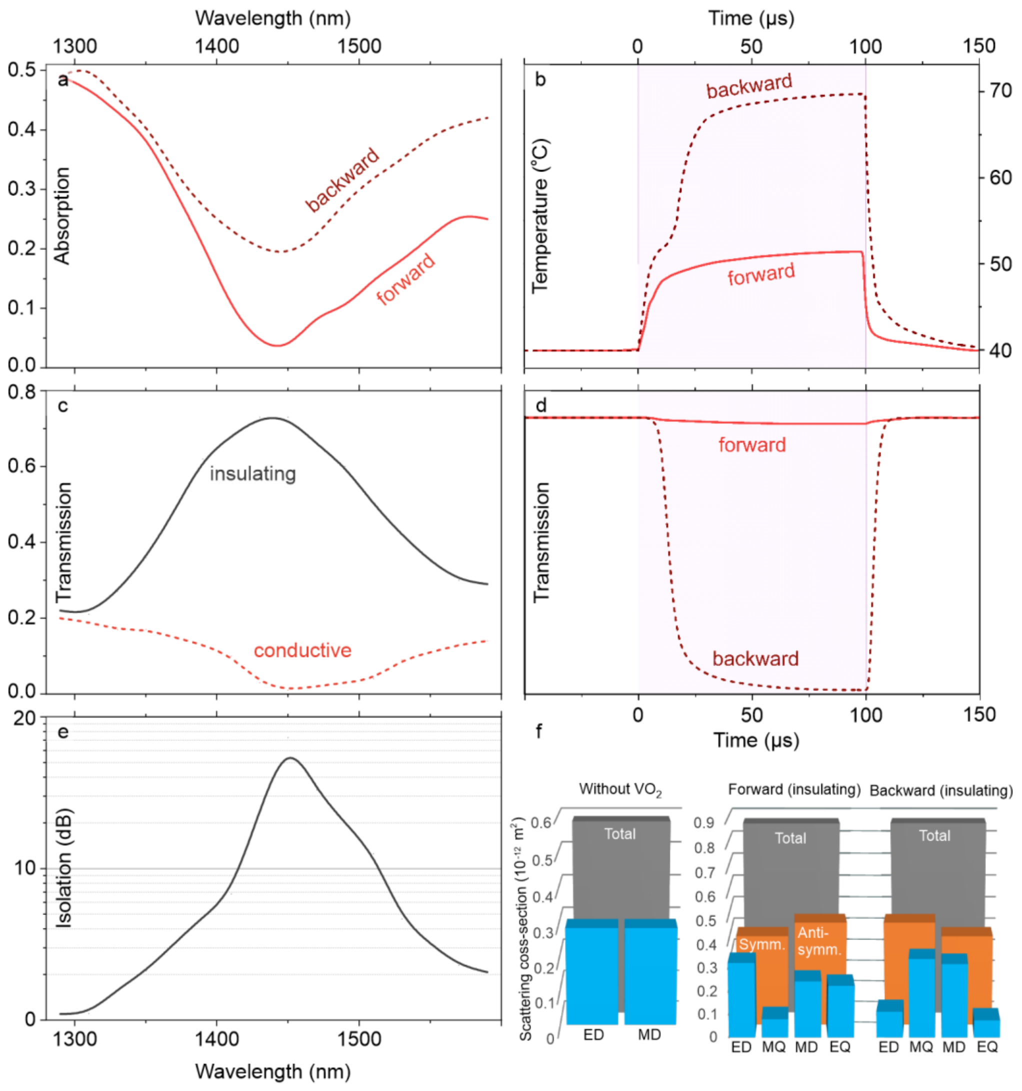

Figure S7. Theoretical study of spectral and temporal response of the nonreciprocal metasurface. (a) Absorption spectra of a Si-VO_2 metasurface for the insulating VO_2 phase and two directions of incidence (forward/backward). (b) Temporal dynamics of VO_2 temperature for the two opposite directions of excitation at 1450 nm wavelength and 200 W/cm^2 power density. (c) Transmission spectra of the metasurface for insulating (black, solid) and conductive (red, dashed) VO_2 phases. (d) Temporal dynamics of the metasurface transmission for the two opposite directions of excitation at 1450 nm wavelength and 200 W/cm^2 power density. (e) Contrast between transmission in insulating and conductive phases. (f) Multipolar composition of the unit cell scattering: (left) metasurface without the VO_2 film featuring identical scattering for the forward and backward directions, (right) with the VO_2 film for the two opposite directions of excitation.

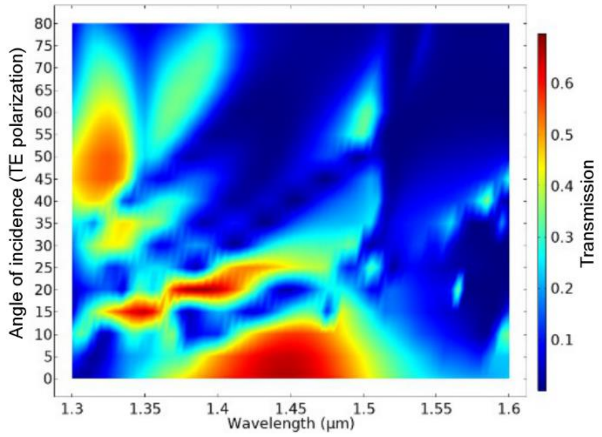

Figure S8. Theoretical analysis of the metasurface transmission vs angle of incidence (cold VO_2)
